# Antiovulatory and estrogenic activity of *Plumbago rosea* leaves in female albino rats

**DOI:** 10.4103/0253-7613.59927

**Published:** 2009-12

**Authors:** E. Sheeja, S.B. Joshi, D.C. Jain

**Affiliations:** Department of Pharmacognosy, B. R. Nahata College of Pharmacy and Research Center, Mhow Neemuch Road, Mandsaur - 458 001, Madhya Pradesh, India

**Keywords:** Antiovulatory activity, estrous cycle, estrogenic activity, antiestrogenic activity, *Plumbago rosea*

## Abstract

**Objective:**

To evaluate the effect of petroleum ether (60–80°), chloroform, acetone, ethanol and aqueous extracts of *Plumbago* rosea leaves on the estrous cycle and to identify the estrogenic activity of active acetone and ethanol extracts in female albino rats.

**Methods:**

Plant extracts were tested for their effect on the estrous cycle at two dose levels: 200 and 400 mg/kg, respectively. The effective acetone and ethanol extracts were further studied on estrogenic activity in rats. Histological studies of the uterus were carried out to confirm their estrogenic activity.

**Results:**

The acetone and ethanol extracts were most effective in interrupting the normal estrous cycle of the rats (*P*<0.05, <0.01, <0.001). These later exhibited prolonged diestrous stage of the estrous cycle with consequent temporary inhibition of ovulation. The antiovulatory activity was reversible on discontinuation of treatment. Both the extracts showed significant estrogenic and antiestrogenic activity.

**Conclusion:**

The acetone and ethanolic extracts of *P. rosea* leaves have an antifertility activity.

## Introduction

Population control is of immense importance for individual and national welfare. Although a variety of synthetic contraceptive agents are available, their use is associated with severe side-effects.[1] Hence, an approach was pursued to identify new antifertility agents from natural sources. Numerous indigenous drugs have been explained in folkloric Indian medicine for the management of various reproduction-related purposes. However, so far, no single plant is available that can be developed into a potent antifertility agent.[2]

*Plumbago rosea* L. (Plumbaginaceae), commonly known as Rakta Chitrak,[3] grows in the wild and in abundance in India. Traditionally, it is used in inflammatory disorders, skin diseases,[4] gastric acidity, constipation, abdominal pain[5] and as an abortifacient.[6–8] The roots of the plant have been reported to possess antitumor[9] and antiatherogenic[10] activities. The active constituents reported in this plant are plumbagin,[11] hydroxy-1,4-napthaquinone, sitosterol glycoside, fatty alcohol and tannins.[12]

So far, during the literature survey, it was found that the roots of this plant have been used for their antifertility and uterine activity.[13] The present study was carried out to evaluate the antifertility effect of *P. rosea* leaves. The uprooting of the plant could be avoided in case the antifertility action was observed.

## Materials and Methods

### Plant material

The leaves of *P. rosea* L. were collected from Kanyakumari district, T.N., and positively identified by Dr. H. S. Chatree, Botanist, Govt. Arts and Science College, Mandsaur, M.P. Voucher specimen (P/002/2006/BRNCOP) was deposited in the herbarium of the Department of Pharmacognosy, BRNCP, Mandsaur.

### Extraction

The leaves of the plant were shade dried and powdered. The powdered material was extracted using petroleum ether (60–80°) for 72 h and successively extracted with chloroform, acetone, ethanol and water for 72 h each in a soxhlet apparatus. The extracts were evaporated under reduced pressure to obtain solid masses and the percentage yield of the extracts was found to be 2.32%, 2.05%, 1.5%, 4.57% and 25.6%, respectively.

### Phytochemical screening

In order to determine the presence of alkaloids, glycosides, flavones, tannins, terpenes, sterols, saponins, fats and sugars, a preliminary phytochemical study (color reactions) with leaf extracts was performed.[14]

### Animals

Female albino rats (Wistar strain weighing 150–200 g) were used for antiovulatory activity and immature female rats (Wistar strain), 21–23 days old, were used for estrogenic activity. The animals were housed in standard environmental conditions of temperature (21 ± 2°C), humidity (55 ± 10%) and a 12-h light–dark cycle. Rats were supplied with standard pellet diet and water *ad libitum*. The animals were acclimatized to laboratory hygienic conditions for 10 days before starting the experiment. Animal study was performed in the Division of Pharmacology, B R Nahata College of Pharmacy, Mandsaur, with due permission from the Institutional Animal Ethics Committee (Reg no. 918/ac/05/CPCSEA).

### Acute toxicity studies

The acute toxicity test of the extracts was determined according to the Organization for Economic Co-operation and Development guidelines no. 420. Female Wistar rats (150–180 g) were used for this study. After the sighting study, a starting dose of 2,000 mg/kg (p.o.) of the test samples were given to various extract groups containing five animals in each groups. The treated animals were monitored for 14 days for mortality and general behavior.

### Antifertility activity

### Antiovulatory activity

Experiments were carried out in female Wistar rats weighing (150–200 g). The vaginal smear of each rat was examined daily between 9–10 A.M for 15 days to select the animals showing regular cycles (4–5 days).[15] The selected rats were divided into 11 groups of six animals each. The extracts were administered orally for five days to cover one regular estrous cycle. Group I received vehicle (1% Tween 80, p.o. daily) and served as control. Groups II to XI received petroleum ether, chloroform, acetone, ethanol and aqueous extracts of *P. rosea* leaves at 200 and 400 mg/kg body weight. Vaginal smear from each animal was observed every morning between 9–10 A.M for five days of treatment and subsequently for 15 days.

### Estrogenic and antiestrogenic activity

The extracts with antiovulatory activity were further studied for the estrogenic and antiestrogenic activity.[16] Immature female Wistar strain rats, 25–30 days old, weighing between 35 and 45 g, were divided into 10 groups of six rats each. The first group served as control and received vehicle only (1% Tween 80). The second group received ethinyl estradiol (standard) in distilled water, 0.02 mg/kg body weight. The third, fourth, fifth and sixth groups received acetone and ethanolic extracts of *P. rosea* leaves at two dose levels, 200 and 400 mg/kg body weight, respectively. The groups VII to X received ethinyl estradiol in addition to a test dose of acetone and ethanolic extract of the plant at the same dose. All the above treatments were given for three days (p.o.). On the fourth day, the rats were sacrificed by decapitation, the uteri dissected out and the surrounding tissues were removed. The uteri were blotted on filter papers and weighed quickly on a sensitive balance and fixed in Bouin's fluid for 24 h. The paraffin-embedded tissues were cut at 6 *μ*m and stained with hematoxylin-eosin solution for histological observations.

### Statistical analysis

The data were statistically analyzed and expressed as mean±SEM. Statistical analysis of the variance between control and experimental values was performed by Student's *t*-test.

## Results

### Phytochemical screening

The phytochemical screening of different extracts of *P. rosea* leaves revealed the presence of various constituents, as shown in Table 1.

**Table 1:** Phytochemical screening of different extracts of *P. rosea*

*Plant extracts*	*Constituents*
Pr-P	Fats, steroids and napthaquinone
Pr-C	Steroids and napthaquinone
Pr-A	Tannins, flavonoids, triterpenoids and napthaquinone
Pr-E	Carbohydrates, glycosides, tannins, flavonoids and saponins
Pr-W	Carbohydrates, glycosides, tannins, flavonoids, proteins and saponins

Pr, *Plumbago rosea*; P, petroleum ether extract; C, chloroform extract; A, acetone extract; E, ethanolic extract; W, aqueous extract

### Acute toxicity studies

No mortality and behavioral changes were observed in the treated groups up to 2,000 mg/kg body weight. The 400 mg/kg dose was chosen as maximum dose for further experiments.

### Effect of extract on the estrous cycle of rats

The present study revealed that the acetone and ethanol extracts of *P. rosea* leaves showed an antifertility effect. Treatment of rats with acetone and ethanolic extracts for five days prolonged the estrous cycle significantly (*P*<0.05, <0.01, <0.001), as indicated in Table 2. The estrous cycle in rats treated with acetone and ethanolic extracts showed reduced duration of estrous and metestrous phases, characterized by a prolongation of the diestrous phase. Withdrawal of the treatment did not indicate any significant change either in the four phases of the estrous cycle or in the duration of the cycle. Acetone extract was found to be more active.

**Table 2:** Effect of *P. rosea* leaf extracts on estrous cycle in rats (values are expressed as mean±SEM, *n*=6)

*Treatment*	*Dose (mg/kg)*	*Duration of cycle (days)*	*Duration of different phases of estrous cycle (days)*			
			
			*Proestrous (days)*	*Estrous (days)*	*Metestrous (days)*	*Diestrous (days)*
Control	---	4.32 ± 0.22	0.83 ± 0.17	0.83 ± 0.17	0.83 ± 0.31	1.83 ± 0.40
Pr. Pet. ether	200	3.66 ± 0.28	0.67 ± 0.33	0.66 ± 0.21	1.00 ± 0.36	1.33 ± 0.21
	400	4.24 ± 0.48	1.00 ± 0.51	0.83 ± 0.30	0.75 ± 0.30	1.66 ± 0.80
Pr. chloroform	200	3.61 ± 0.35	0.50 ± 0.22	0.66 ± 0.21	0.95 ± 0.45	1.50 ± 0.50
	400	3.78 ± 0.52	0.67 ± 0.33	0.66 ± 0.66	0.91 ± 0.27	1.33 ± 0.84
Pr. acetone	200	5.75 ± 0.49**	0.26 ± 0.21*	0.83 ± 0.65	0.66 ± 0.33	4.00 ± 0.51**
	400	5.52 ± 0.37***	0.33 ± 0.21*	0.50 ± 0.24	0.86 ± 0.27	3.83 ± 0.65***
Pr. ethanol	200	5.15 ± 0.38*	0.33 ± 0.21*	0.66 ± 0.49	0.83 ± 0.27	3.33 ± 0.55*
	400	5.20 ± 0.47*	0.46 ± 0.31	0.66 ± 0.33	0.75 ± 0.44	3.33 ± 0.80**
Pr. aqueous	200	4.07 ± 0.41	0.66 ± 0.33	1.00 ± 0.44	1.08 ± 0.32	1.33 ± 0.61
	400	4.57 ± 0.49	0.50 ± 0.50	0.66 ± 0.21	1.08 ± 0.41	2.33 ± 0.84

Pr, *P. rosea*.

* *P*<0.05

** *P*<0.01

*** *P*<0.001 vs. control (Student's t-test)

### Estrogenic and antiestrogenic activity

**Figure 1:**
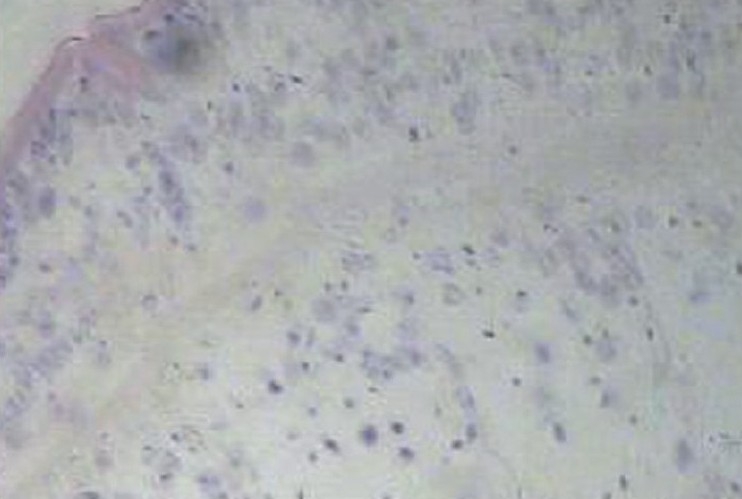
Photomicrograph of a transverse section of the uterus of control rats

**Figure 2:**
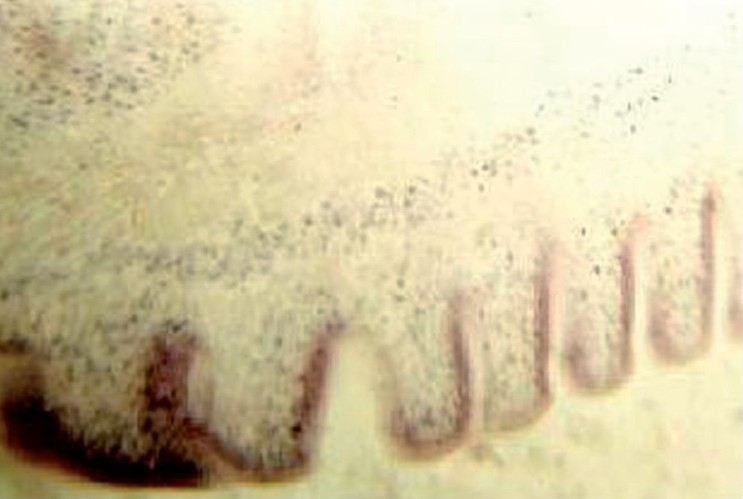
Photomicrograph of a transverse section of the uterus of ethanol extract, 400 mg/kg p.o-treated rats, with increase in the thickness of the endometrium

**Figure 3:**
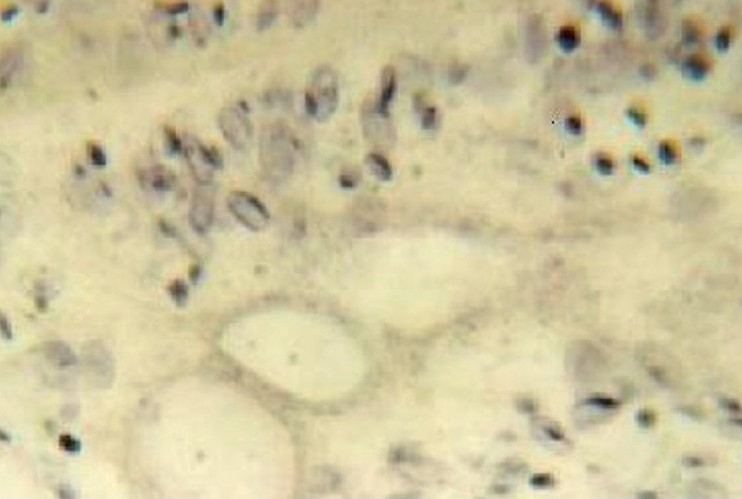
Photomicrograph of a transverse section of the uterus of acetone extract at 400 mg/kg p.o -treated rats with stroma consisting of loose fibrous tissues with edema

The effect of both acetone and ethanolic extracts of *P. rosea* leaves on immature rat uterus is shown in Table 3. Oral administration of the extracts at 200 and 400 mg/kg body weight caused a significant increase in the uterine weight in immature rats (*P*<0.05, <0.01, <0.001). The thickness of the endometrium was significantly increased when compared to the control rats [Figures 1 and 2]. The endometrium epithelium consisted of spindle-shaped cells with basal nuclei and the endometrial glands were dilated. The stroma consisted of loose and edematous fibroblast-type cells with edema [Figure 3]. The control rats showed closed vagina whereas the treated rats showed an open vagina.

**Table 3:** Estrogenic and antiestrogenic activity of acetone and ethanol extracts of *P. rosea* leaves. Values are expressed as mean ± SEM, *n*=6

*Groups*	*Dose (mg/kg body weight)*	*Uterine weight (mg/100 g body weight)*
Control	(Tween 80, 1%)	45.50 ± 2.27
Ethinyl estradiol	0.02	142.00 ± 4.83***
Pr. acetone	200	79.33 ± 5.81**
Pr. acetone	400	82.00 ± 5.15***
Pr. ethanol	200	68.00 ± 3.86***
Pr. ethanol	400	75.33 ± 6.15***
Ethinyl estradiol + Pr. acetone	0.02 + 200	123.33 ± 7.96***†
Ethinyl estradiol + Pr. acetone	0.02 + 400	131.83 ± 4.32***†
Ethinyl estradiol + Pr. ethanol	0.02 + 200	125.17 ± 3.65***†
Ethinyl estradiol + Pr. ethanol	0.02 + 400	120.07 ± 7.41***†

Pr, *P. rosea*

** *P*<0.01

*** *P*<0.001 vs. control

† *P*<0.05 vs. ethinyl estradiol (Student's t-test)

**Figure 4:**
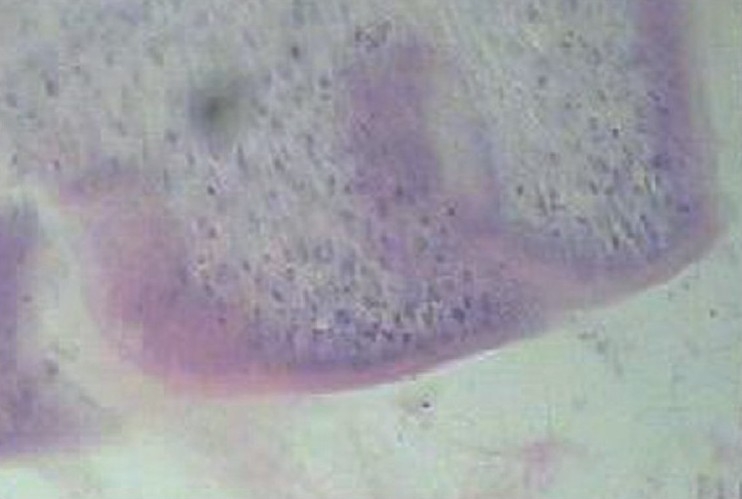
Photomicrograph of a transverse section of the uterus of ethinyl estradiol (0.02 mg/kg p.o)-treated rats showing proliferation stage.

The administration of acetone and ethanol extracts aggravated a significant increase in the uterine wet weight, signifying the estrogenic activity. However, when treated with ethinyl estradiol, it lowered the effect of estrogenic activity produced by ethinyl estradiol alone [Figure 4, Table 3]. Comparatively, the acetone extract was found to be more active.

## Discussion

**Figure 5:**
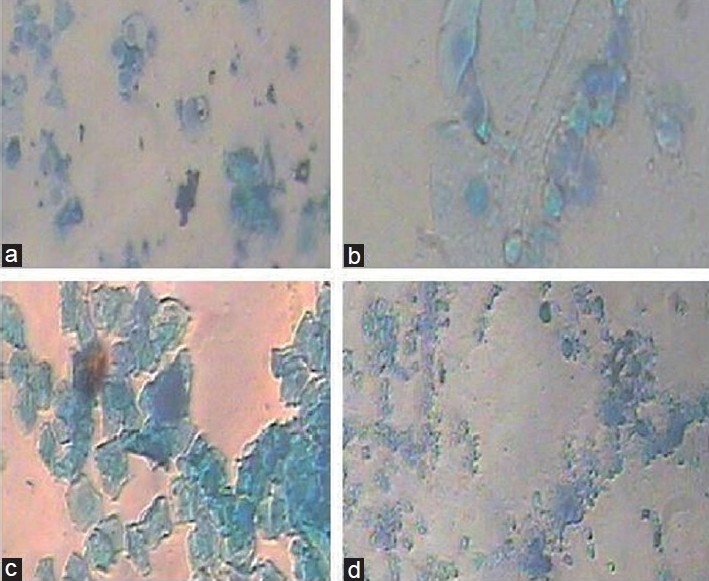
Vaginal smear of the rats on four-day estrous cycle. CXR III camera × 400. Control: 1st day (A), 2nd day (B), 3rd day (C), 4th day (D)

The acetone and ethanolic extracts of *P. rosea* leaves exhibited significant (*P*<0.05, *P*<0.01, <0.001) antifertility activity. The duration of the estrous cycle in rats is normally 4–5 days. Three cell types are found in the vaginal smear during a normal rat estrous cycle. The presence and absence of these cell types, and the relative proportion of each cell type, determine the stages of the estrous cycle [Figure 5]. Of the five extracts tested for the antiovulatory activity, acetone and ethanol extracts produced a temporary and reversible modification on the estrous cycle. The prolongation in the diestrous phase explains the remote possibility of the rats getting pregnant. The reversible nature of the antifertility activity of the extract is explained through the observation that there was no significant change in the diestrous and the estrous cycle after withdrawing the extract from those of the control. As a result, the extracts provoked inhibition of the ovulation with consequent reduction of the cyclicity. Estrous cycle and the shift in different stages are mainly governed by the synthesis of ovarian estrogen, which, in turn, is controlled by the secretion of pituitary gonadotropins and hypothalamic-releasing factor.[15]

The extracts with the antiovulatory activity were further studied for their estrogenic and antiestrogenic activity. These extracts also exhibited estrogenic activity as shown by the significant increase in the diameter of the uterus, uterine weight and thickness of the endometrial epithelium when compared with the control. It was also observed that the acetone and ethanol extracts suppressed the action of ethinyl estradiol when administered together. The extracts showed a significant estrogen-like activity when given alone but, with ethinyl estradiol, they exhibited a slight antiestrogenic nature. This indicates that the extract acted as a competitive antagonist to the more potent ethinyl estradiol.[17]

Preliminary phytochemical studies indicated the presence of tannins, flavonoids, triterpenoids and napthaquinone in the acetone extract and the ethanol extract showed the presence of carbohydrates, glycosides, tannins, flavonoids and saponins. According to the literatures, flavonoids and plumbagin (napthaquinone) are known to exhibit antifertility activity.[1,17,18] In our study, it is not solely the presence of napthaquinones or flavonoids that produced the antifertility activity because the petroleum ether extract contains napthaquinones and was devoid of any activity. On the other hand, irrespective of the absence of napthaquinones, the ethanol extract displayed significant activity when compared with controls, indicating that flavonoids could be responsible for the activity. The synergism produced by napthaquinones along with flavonoid could be the reason for the enhanced activity of the acetone extract when compared with that of the ethanol extract.

## Conclusion

The results of the present study indicate that the acetone and ethanolic extracts of *P. rosea* leaves have significant antifertility activity. The leaves of this plant could be used to induce abortion. The extracts of this plant can be further explored for contraceptive use.
